# “Feelings and Fitness” Not “Feelings or Fitness”–The *Raison d'être* of Conservation Welfare, Which Aligns Conservation and Animal Welfare Objectives

**DOI:** 10.3389/fvets.2018.00296

**Published:** 2018-11-27

**Authors:** Ngaio J. Beausoleil, David J. Mellor, Liv Baker, Sandra E. Baker, Mariagrazia Bellio, Alison S. Clarke, Arnja Dale, Steve Garlick, Bidda Jones, Andrea Harvey, Benjamin J. Pitcher, Sally Sherwen, Karen A. Stockin, Sarah Zito

**Affiliations:** ^1^Animal Welfare Science and Bioethics Centre, School of Veterinary Science, Massey University, Palmerston North, New Zealand; ^2^Centre for Compassionate Conservation, School of Life Sciences, University of Technology Sydney, Sydney, NSW, Australia; ^3^Wildlife Conservation Research Unit, Department of Zoology, Recanati-Kaplan Centre, University of Oxford, Oxfordshire, United Kingdom; ^4^Institute of Land Water and Society, Charles Sturt University, Albury, NSW, Australia; ^5^Veterinary Emergency Centre and Hospital, JCU Vet, James Cook University, Townsville, QLD, Australia; ^6^Royal New Zealand Society for the Prevention of Cruelty to Animals, Auckland, New Zealand; ^7^Possumwood Wildlife Recovery and Research, Bungendore, NSW, Australia; ^8^Royal Society for the Prevention of Cruelty to Animals Australia, Canberra, ACT, Australia; ^9^Taronga Conservation Society Australia, Sydney, NSW, Australia; ^10^Zoos Victoria, Melbourne, VIC, Australia; ^11^Coastal Marine Research Group, Institute of Natural and Mathematical Sciences, Massey University, Auckland, New Zealand

**Keywords:** conservation welfare, animal welfare assessment, wildlife conservation, environmental ethics, wild animal welfare

## Abstract

Increasingly, human activities, including those aimed at conserving species and ecosystems (conservation activities) influence not only the survival and fitness but also the welfare of wild animals. Animal welfare relates to how an animal is experiencing its life and encompasses both its physical and mental states. While conservation biology and animal welfare science are both multi-disciplinary fields that use scientific methods to address concerns about animals, their focus and objectives sometimes appear to conflict. However, activities impacting detrimentally on the welfare of individual animals also hamper achievement of some conservation goals, and societal acceptance is imperative to the continuation of conservation activities. Thus, the best outcomes for both disciplines will be achieved through collaboration and knowledge-sharing. Despite this recognition, cross-disciplinary information-sharing and collaborative research and practice in conservation are still rare, with the exception of the zoo context. This paper summarizes key points developed by a group of conservation and animal welfare scientists discussing scientific assessment of wild animal welfare and barriers to progress. The dominant theme emerging was the need for a common language to facilitate cross-disciplinary progress in understanding and safeguarding the welfare of animals of wild species. Current conceptions of welfare implicit in conservation science, based mainly on “fitness” (physical states), need to be aligned with contemporary animal welfare science concepts which emphasize the dynamic integration of “fitness” and “feelings” (mental experiences) to holistically understand animals' welfare states. The way in which animal welfare is characterized influences the way it is evaluated and the emphasis put on different features of welfare, as well as, the importance placed on the outcomes of such evaluations and how that information is used, for example in policy development and decision-making. Salient examples from the New Zealand and Australian context are presented to illustrate. To genuinely progress our understanding and evaluation of wild animal welfare and optimize the aims of both scientific disciplines, conservation and animal welfare scientists should work together to evolve and apply a common understanding of welfare. To facilitate this, we propose the formal development of a new discipline, Conservation Welfare, integrating the expertise of scientists from both fields.

## Introduction

Conservation biology and animal welfare science are both multi-disciplinary fields that use scientific methods to address concerns about animals (1, 2). Both also require decision-making in complex ethical milieu and in the face of significant uncertainty (3, 4). While animal welfare science has traditionally focussed on the welfare of domestic animals living under human care, there is increasing recognition of the potential for human activities to also impact on the welfare of wild animals (5, 6). In particular, various human activities aimed at conserving populations, species, ecosystems and, ultimately, biodiversity can influence the welfare of individuals and groups of wild animals (4, 7, 8).

Briefly, there is growing evidence of animal welfare impacts associated with *in situ* conservation activities, such as habitat management, field research, and management of rare and overabundant native animals, as well as, of invasive species [e.g., (9–27)]. Likewise, *ex situ* conservation activities including captive breeding, holding animals indefinitely in zoos as “insurance populations,” wildlife rescue and rehabilitation, reintroductions and research on captive animals can influence animal welfare [e.g., (2, 13, 28–37)].

Such conservation activities are strongly supported by many in society, reflecting the value placed on concepts, such as “naturalness” and “biodiversity,” the continuing existence of current species and retention of “evolutionary potential” (38–41). However, activities impacting detrimentally on the welfare of individual animals may ultimately threaten their survival and fitness and thus the viability of valued populations and species [e.g., (2, 9, 12, 15, 21, 34, 42, 43)], thereby negating some of the intended conservation benefits (3). In addition, growing public awareness of, and concern about, the welfare of individual wild animals necessitates improved transparency and justification of conservation activities (1, 3, 41, 43–46). Thus, the growing urgency for conservation brings with it an equally urgent need for conservation and animal welfare scientists to engage in genuine discourse in support of collaborative research to underpin welfare-focused conservation practices.

Animal welfare is a difficult concept to define but the term is now widely used to reflect how an animal is experiencing its life (47, 48). Dominant theoretical models for understanding animal welfare have focussed on the animal's physical state or biological function (Biological function orientation), the mental experiences, both positive and negative, the animal may have as a result of its physical state/biological function (Affective state orientation) or the naturalness of its environment and/or its ability to express natural behaviors (Naturalness or Natural living orientation) (49, 50). It is now widely agreed within the field of animal welfare science that no single orientation on its own is sufficient and that components of all three theories must be integrated to holistically understand and scientifically assess animals' welfare states (48, 50, 51). Further discussion and illustrations of the limitations of focussing on only one aspect of animal welfare, in the context of conservation, are presented below in The Need for Common Language and Understanding Relating to Wild Animal Welfare.

Conflicts between those working to achieve the goals of conservation biology and those aiming to safeguard the welfare of individual wild animals are apparently on the rise (20, 45, 52). As noted, this may be because of the growing urgency and thus volume and range of conservation research and practices, as well as growing public awareness of conservation activities (3, 12, 43) and, more generally, of animal welfare [e.g., (53–56)]. This is exemplified by the moratorium imposed by the Tasmanian state government in 2000 on hot-branding as a method for identifying individual elephant seals (*Mirounga leonina*) for research purposes on Australia's Macquarie Island after media attention and public outcry about perceived animal welfare impacts (3, 57). In the scientific arena, growing concerns about the effects of conservation activities on wild animal welfare may also be attributed to our increasingly detailed, robust and evidenced-based understanding of what animal welfare is and how it can be evaluated (48, 58–62) (see below).

Such conflicts have often been attributed to incompatible ideologies [e.g., (1, 38, 52, 63–65)]. For example, McMahon et al. (20) suggested that prioritizing concerns for the welfare of individual animals, as “animal welfare advocates” seek to do, stymies the generation of scientific knowledge critical to stemming the extinction of species and the consequent loss of ecosystem services and evolutionary potential. However, the position often cited for “animal welfare advocates” is actually one of animal rights, an ethical stance that no amount of benefit from conservation activities can justify any level of individual animal suffering [e.g., (20, 39, 66, 67)].

In contrast, the role of animal welfare scientists in the conservation context is to use scientific principles and methods to evaluate impacts on the welfare of animals, both positive and negative and at individual and population levels, to inform ethical conservation decision-making and practice (42, 68–70). Accordingly, they advocate approaches to achieving conservation goals that minimize negative welfare impacts [e.g., (64, 70–72)] and, where appropriate and possible, realize or maximize any welfare benefits (44, 46, 61). In some cases, animal welfare scientists may use the outcomes of scientifically robust evaluations to recommend that an activity not proceed if the predicted or actual welfare costs are considered to outweigh the likely conservation benefits (42, 44, 70, 73, 74). For example, application of an identification marking method that would cause significant tissue injury, pain or behavioral alteration and that would not facilitate animal identification at a level (individual or group) or distance or for a duration required to achieve the objectives of the research programme would be discouraged (44).

This kind of informed decision-making is equally recommended by conservation scientists [e.g., (2, 20, 21, 43, 75, 76)]. Thus, the starting positions and goals of conservation biology and animal welfare science do not appear to be inherently incompatible. Given that activities impacting detrimentally on the welfare of individual animals often also hamper achievement of some conservation goals (2, 43, 77) and that societal acceptance is imperative to the continuation of conservation activities (3, 20, 44), it is clear that the best outcomes for both disciplines will be achieved through collaboration and knowledge-sharing. Despite this recognition, cross-disciplinary information-sharing and collaborative research and practice in conservation are still relatively rare [e.g., (2, 20, 34, 78)], with the exception of the zoo animal context (see below), so that substantial scope for synergy between the activities of conservation and animal welfare scientists remains.

The aim of this paper is to summarize key points developed by a group of conservation and animal welfare scientists discussing scientific assessment of wild animal welfare. On the basis of those discussions, we propose the formal development of a new discipline, integrating the expertise of scientists from both fields, to progress our understanding and evaluation of wild animal welfare and optimize the aims of both disciplines: this is “Conservation Welfare,” an appellation coined in the World Association of Zoos and Aquariums' Animal Welfare Strategy document in 2015 (46).

## Participants and workshop

Workshop participants were invited from those attending the third International Compassionate Conservation Conference in Sydney, Australia in November, 2017. The over-arching purpose of the 1-day workshop was to explore the various roles of science in “Conservation Welfare.” Fourteen participants from Australia, New Zealand and the United Kingdom attended, and the workshop was facilitated by the two lead authors. The participants were animal welfare scientists, conservation scientists, scientific representatives of non-governmental animal welfare organizations, wildlife veterinarians and wildlife rehabilitators.

The workshop comprised a series of group discussions exploring the meaning of animal welfare and how it might be assessed, as well as, the ways in which conservation activities might impact upon wild animal welfare. In addition, the challenges associated with understanding wild animal welfare and integrating that kind of understanding into conservation policy and practice were explored. Following the workshop, the lead authors distilled from those discussions key principles for optimizing the aims of both scientific disciplines. The dominant theme emerging from the workshop was the need for a common language to facilitate cross-disciplinary progress in understanding and safeguarding the welfare of animals of wild species.

## The need for common language and understanding relating to wild animal welfare

There are two key reasons why conservation and animal welfare scientists should work together to evolve and apply a common understanding of welfare as it pertains to animals of wild species. First, the way in which animal welfare is conceived influences the way it is evaluated and the emphasis put on different features of welfare. This is important because rigorous, defensible and transparent assessment of “animal suffering” is key to making informed and ethical decisions in conservation practice (2, 16, 20, 69, 70). Secondly, the conception of animal welfare influences the importance placed on the outcomes of such evaluations and how that information is used going forward, for example, in policy development and decision-making.

## Conception of animal welfare influences its evaluation and emphasis–“fitness”

The theoretical characterization of animal welfare directly influences both the approach to its assessment and the dimensions or features emphasized in such evaluations. Specifically, what welfare is considered to be dictates the indicators measured, the level of measurement (e.g., individual vs. population level), the aspects of welfare prioritized and how the data are interpreted (61). This can be illustrated by examining the apparently different characterizations of welfare in conservation and animal welfare sciences and the practical implications of these differences.

Logically, current conceptions of welfare in conservation biology often appear to align to the immediate goals of the discipline, that is, to keep genetically valuable individuals alive and reproducing and to maintain genetic diversity within and between populations [e.g., (21, 26)]. In accordance with this, welfare is often evaluated at the population level and using variables chiefly related to the physical state or biological function of the animals. At the most general level, welfare may be extrapolated from measures of survival and reproductive success, i.e., “*fitness”* [e.g., (79–86)].

Other conservation evaluations focus on variables that reflect the animals' physiological or health status in finer detail, i.e., specific attributes of their “*fitness*,” and may be undertaken at the population or individual level, depending on the purpose and on practical considerations (44). Examples include body condition, weight, coat, plumage or skin condition, injury or pathology, altered gait or the occurrence of abnormal behaviors [e.g., (26, 84, 87–90)]. Likewise, blood, saliva and fecal components indicating nutritional status or energy reserves, immune or reproductive function or “stress” may be evaluated [e.g., (13, 91–94)]. In rehabilitation, translocation and reintroduction contexts, when animals are under closer human control for longer periods, clinical examinations may be performed to evaluate the health status and potential survivability of rescued, captured or captive wild animals [e.g., (35, 95–100)]. Similarly, in the research context, the impacts of manipulations, such as identification marking or capture are commonly evaluated using measures of physical status, such as injury severity or healing, changes in body weight, condition or temperature, energy expenditure, behavior or survival estimated by likelihood of re-sighting/recapture [reviewed by (11, 22, 101–106)].

More detailed evaluations of wild animal behavior are generally undertaken to understand features of the social and ecological interactions of animals, as well as the impacts of human interventions or changes to the ecosystem on fitness and ecosystem function, rather than to understand their welfare state *per se* [e.g., (2, 9, 34, 107–112)]. Notable exceptions are the detailed studies of behavior often undertaken in the zoo context for the explicit purpose of assessing welfare state [e.g., (113–116)] and systematic evaluations of wild behavior to improve the efficacy of strategies to control invasive species [e.g., reviewed by (117–119)].

### Conception of animal welfare influences its evaluation and emphasis–“feelings”

In the field of animal welfare science, welfare is generally conceptualized as a property of an individual animal. More specifically, welfare is a property of individuals of species considered to have the capacity for both pleasant and unpleasant mental experiences, i.e., experiences that matter to the animal itself; this capacity is otherwise known as sentience (58, 120–122). Such experiences are generated by processing of information about the animal's internal physical state and/or its external circumstances and are variously called affects, affective states, emotions or “feelings” (48, 51). Thus, while welfare can be assessed at the population level (as routinely occurs in assessment of farm animal welfare), the underlying assumption is that population-level indicators reflect the mental experiences of the various individuals within the group (62, 123, 124), rather than a population collectively possessing welfare *per se*.

In accordance with this conceptualization, there is now wide acknowledgment in this scientific field of the importance of animals' mental experiences as the feature of ultimate relevance for understanding their welfare (48, 58, 121, 122, 125). Related to this is recognition of the importance of assessing the potential for both negative (unpleasant) and positive (pleasant) experiences to holistically understand welfare state at any point in time (58, 59, 61, 62, 126). Thus, animal welfare evaluations aim to interpret the indicators of physical/functional state, i.e., biological function or “fitness,” in terms of the mental experiences that those indicators are likely to reflect, i.e., “feelings.”

In support of this approach, there is growing understanding of the neurophysiological bases of mental experiences, such as thirst, hunger, pain, breathlessness, nausea, fear and others, as well as evidence of the links between measurable indicators of physical/functional states and the occurrence of such mental experiences in some non-human animals [e.g., (126–132)]. Importantly, affective states can also influence physical/functional states; thus the two are inextricably and dynamically inter-related and should be interpreted as such (48, 50, 133). For example, it is well-established that dairy cattle, pigs and poultry which are more fearful of their human handlers exhibit lower productivity and/or reproductive success than their less fearful cohorts (134). This advancing biological understanding and evidence facilitates cautious interpretation of the kinds of data already collected in some conservation research as reflecting the mental experiences of the animals and thus their welfare state, e.g., hydration status or changes in body condition (94) as indicators of thirst and hunger, respectively (132).

### Conception of animal welfare influences its evaluation and emphasis–“feelings” and “fitness”

Framed in this way, the limitations of using survival and reproductive success as proxies for welfare are clear. Simply surviving until the point of evaluation does not guarantee acceptable or desirable welfare, as animals can survive despite experiencing chronic unpleasant states (13, 135–138). This recognition may influence decisions between lethal and non-lethal population control strategies or attempts at rehabilitation and release vs. euthanasia for rescued wildlife [e.g., (15, 17, 18, 23, 24, 28, 35, 139, 140)]. Likewise, measures of survival and reproductive fitness are not useful for evaluating welfare impacts when animals are intentionally killed for conservation purposes [lethal control of invasive species or culling overabundant or nuisance native animals: e.g., (18, 25, 32, 73, 141–143)], or when they die due to unintended effects of conservation activities [e.g., (9, 15, 72)].

Alternatively, although low reproductive success might reflect physiological states that align with poor welfare, such as malnutrition or severe stress [e.g., (13, 15, 17, 144)], failure to reproduce, *per se*, is not necessarily indicative of a specific negative experience that would compromise welfare (4) and vice versa (137). This point might be important when considering the welfare both of valued animals that are not reproducing [e.g., cheetahs in captivity; (145, 146)] and when reproductive control is used to manage wild populations [e.g., (23, 147)].

Likewise, a sole focus on biological function can lead to interpretation of “normal” health or function as sufficient evidence of good or acceptable welfare or lead to emphases on “inputs” (i.e., good husbandry or care) that may not translate into acceptable “outputs” (i.e., good welfare) (34, 46, 133, 148). To illustrate these risks, many domestic farmed animals have good biological function and are highly productive, in terms of survival, growth and reproduction, but have poor welfare due to limited opportunities for normal behavioral expression and the attendant unpleasant mental experiences (135, 136, 149). Healthy wild animals may have unpleasant experiences too. Examples include significant anxiety or fear during capture, captivity or after transfer to a new location or social group (2, 12, 34, 125), or less well-understood experiences, such as loneliness, boredom or frustration in captive environments (61, 150, 151). Focussing only on indicators of physical status or biological function can also result in failure to look for or recognize indicators of the wide variety of unpleasant experiences that can compromise welfare (48, 152). Related to this, there is also a danger that the theoretical underpinnings of welfare evaluations may be conceived, *post-hoc*, to fit the limited data that can currently be collected in practice, rather than the preferred strategy of the established conceptual framework of welfare guiding the approaches to data collection and the identification of gaps to advance knowledge for future assessments (153).

In the context of killing, a focus on biological function may lead to over-estimates of welfare impacts. One commonly held view amongst animal welfare scientists is that death *per se* does not equate to poor welfare [cf. (154)]; an animal's experiences of its welfare state exist only while it is alive and able to consciously perceive features of its internal state and/or the world around it (42, 48, 51). Thus, negative welfare impacts take the form of unpleasant experiences, such as pain, breathlessness, nausea or fear before the irreversible loss of consciousness [i.e., the point at which experiences are no longer possible (152)]. Measures of physical state (i.e., behavior or physiology) made after this point no longer reflect conscious mental experiences and, although they are often aesthetically unpleasant to observe, they do not reflect welfare state (25, 32).

### Previously proposed concepts to unite conservation and animal welfare sciences

Several authors have previously indicated the need for a common language to unite conservation and animal welfare sciences and have attempted to identify common metrics to do so and to more clearly delineate the point at which biological fitness and welfare converge. “Stress” was proposed as that unifying concept, and measurements of stress have been widely used to evaluate the fitness and welfare impacts of human-generated conditions and procedures on animals of wild species [e.g., (42, 75, 78, 91, 92, 94, 155, 156)]. Stress has usually been characterized according to physiological responses, primarily activation of the hypothalamic-pituitary-adrenal (HPA) axis elicited by external threats or disruptions to internal conditions, i.e., homeostasis (157, 158).

As such, measurements of stress are often used to infer how well the animal is “coping” with its environment (159). But the affective significance of such stress, coping or lack of coping and thus the relationship to welfare state, is not clear (160). For instance, “stress responses” can also occur in situations actively sought out by animals and which would intuitively appear to be related to positive experiences e.g., hunting, mating (91). In addition, the responses and responsiveness of the HPA axis can change depending on the pattern and duration of the stressors [e.g., (92, 113, 161)], stress may have negative (e.g., reduced reproduction) or positive (e.g., escaping a predator) consequences for fitness (75), and behavioral strategies may be used instead to “cope” with conditions that are nonetheless unpleasant to the animal, e.g., hiding, expressing abnormal repetitive behaviors (114, 162).

In response to these limitations of using “stress,” several authors proposed “distress” as the point at which physiological stress becomes intense and/or prolonged enough to be detrimental to both welfare and fitness (75, 113). Distress is variously defined as “a chronic condition reflecting the biological cost of repeated or cumulative stressors” (157) or “when stress induces allostatic [homeostatic] overload or becomes pathogenic” (163). So defined, distress reflects some point toward the extreme end of the continuum of physiological stress; the point at which stress becomes distress is empirically identified as when diversion of resources away from core functions, such as reproduction, feeding or immune function can be quantified (13, 157, 164). The concurrent measurement of stress (i.e., HPA activation) and biological cost (e.g., suppressed reproductive function) makes this concept valuable for assessing the conservation implications of stressors that may also impact on welfare (13, 137).

In contrast, in the field of animal welfare science, distress is generally characterized as “one or more negative psychological states indicative of poor wellbeing or that decrease wellbeing” (165) or as a “wide range of unpleasant emotional experiences” (166). Thus, distress in this field unequivocally represents the extreme end of a continuum describing affective, mental or psychological states while stress (and distress in the conservation context) appears primarily to represent a physiological response, with ambiguous relationships to affective state. Accordingly, these concepts do not occupy the same continuum. An important corollary of this is that the absence of evidence of extreme stress responses and/or fitness costs is not evidence of the absence of unpleasant experiences and poor welfare state.

This affect-related concept of distress is more consistent with the current conception of welfare favored by the majority of animal welfare scientists (48). However, given that distress is characterized as a range of different unpleasant experiences and that different mental experiences reflect different problems for the animal to solve via their behavioral and physiological responses (61), there is unlikely to be one single empirical metric of both reduced fitness and poor welfare nor even a single set of measurable indicators that can be used to practically evaluate distress (167). It is more meaningful to evaluate welfare according to the evidence about the intensity and duration of specific unpleasant experiences, such as breathlessness, pain, thirst and hunger (62). Doing so also facilitates the development and implementation of strategic approaches to avoiding or mitigating those specific experiences (152).

Thus, the problem with concepts, such as stress, distress and others like “suffering” is the lack of clarity about their meaning and their relationships to the mental experiences of animals and the associated lack of a scientific framework for assessing these scientifically nebulous concepts (51, 168). Interestingly, although such pragmatic models have been advocated for more than 15 years, there has been limited uptake in practice, and collaborative research and activity between animal welfare and conservation scientists is still rare (2). Perhaps this is because, more fundamentally, a common understanding of what animal welfare is conceived to be must be achieved first.

## Characterization of welfare also influences the significance assigned to, and thus the application of, outcomes of welfare assessments

As well as influencing the approach to, and emphasis within, scientific assessments, the conceptual foundations of welfare influence the ways the outcomes of those assessments are interpreted, prioritized and applied. Specifically, how welfare is understood may influence the following: decisions about whether welfare is assessed at all; how strongly minimization of negative welfare impacts is emphasized; how information from welfare assessments is integrated into conservation decision-making; and how that knowledge informs the development of policies, guidelines and legislation. Salient examples from the New Zealand (NZ) and Australian context are presented below.

Overall, it is argued that understanding welfare as what is experienced by, and thus what matters to, the animal itself increases our responsibility in three areas: to systematically evaluate welfare impacts; to genuinely include that knowledge in conservation decision-making practice; and to give it more appropriate prominence in those decisions than is currently apparent (52).

### Whether or not to devote resources to welfare assessment and how strongly minimization of negative welfare impacts is emphasized

Many kinds of conservation activities proceed without explicit or formal scientific evaluation of potential welfare impacts. In NZ, these include routine management of threatened native animals (such as captive breeding and release, intensive monitoring, and regular movement between populations), control of invasive animal populations, wildlife rescue and rehabilitation, and permanently holding native and exotic wild animals in captivity. Decisions about whether to undertake formal welfare assessment may be made implicitly or explicitly by various stakeholders with various objectives; such decisions may sometimes involve conflicts of interest, i.e., not wanting to know about the welfare impacts of activities considered to be desirable for other reasons, including for the achievement of conservation objectives. While it may be argued that many such activities are “routine” or based on “best practice,” the lack of ongoing welfare assessment limits opportunities to update practices as scientific understanding and technical capacity grow (169), thereby limiting opportunities to minimize negative welfare impacts.

Characterization of welfare may also influence the emphasis put on minimizing negative impacts in the context of conservation research. In NZ, research on wild animals must be approved by animal ethics committees (AECs) authorized under the Animal Welfare Act (1999) (170); approval depends on demonstrating an understanding of the potential negative impacts on the subject animals' welfare as well as the likely benefits of the research. However, there may be unrealized opportunities for minimizing welfare impacts associated with research procedures, and it behooves AECs and the applicants seeking approval to regularly challenge the status quo in terms of what might be considered to be “unavoidable” negative welfare impacts. As a parallel, while surgical procedures performed on laboratory animals almost inevitably cause some degree of pain, NZ AECs put the onus on applicants to demonstrate how such pain can be minimized and that pain relief strategies are the best currently available [e.g., (153, 171, 172)]. Likewise, academic journals in the field of animal welfare science are increasingly demanding evidence, above and beyond appropriate regulatory approval, of strategies to avoid, mitigate or minimize negative welfare impacts on research animals [e.g., Animal Welfare journal; (43)].

To better realize these sorts of opportunities, research directed at minimizing existing welfare impacts associated with conservation activities should be encouraged and specifically funded (153). As one example, systematic evaluations of the effects of identification marking techniques on the welfare of subject animals are still rare relative to the number of studies applying such techniques to wild populations [e.g., reviewed by (11, 22, 101, 103)], and more are needed (169). Whenever the type, severity, duration, distribution or variability of negative welfare impacts are not well-understood, preliminary studies that formally assess the impacts of the proposed procedures should be required by AECs before granting approval for major studies applying those procedures in wild populations (44, 153, 169).

## Whether and/or how to integrate information from assessments into decision-making

In line with the points made above, decisions about whether and how a wider range of conservation activities proceed should be informed by impacts on the animals involved (20). As noted, such decisions are complex and involve multiple stakeholders with differing priorities [e.g., (1, 45)]. However, such decisions cannot be taken knowledgeably and ethically if welfare impacts are not rigorously and transparently evaluated (72, 74). Assessments that emphasize the importance of mental experiences to an animal's welfare and that cautiously interpret measured physical/functional variables accordingly may result in greater weight being given to the welfare outcomes in conservation decision-making. Alternatively, there is a risk that evaluations focussing only on “objective” clinical indicators of biological function will inspire less concern for animal subjects of conservation activities, leading to prioritization of other objectives in conservation decision-making.

To illustrate, despite rigorous scientific research demonstrating the negative experiential impacts of poisons used to lethally control various invasive mammal species in NZ and Australia [e.g., (18, 25, 32, 173, 174)], both small-scale domestic applications (e.g., household rodent control) and mass poisoning programmes continue to use the least humane agents because they are effective and safe for humans (118, 175). In the last 30 years, relatively little progress has been made toward developing effective and safe alternatives that are demonstrably more humane for the millions of sentient animals so affected (175, 176). Perhaps explaining those welfare impacts in terms of the severely unpleasant and protracted experiences that the animals may have before loss of consciousness (25, 32, 174) would influence the weight assigned to welfare when deciding to continue to use those agents.

Framing welfare impacts in terms of the unpleasant experiences animals might have may therefore also be useful for informing public sentiments and political decisions regarding lethal vs. non-lethal control of both native and introduced species. With regard to non-lethal methods, wild animals clearly demonstrate species-specific indicators of experiences, such as extreme fear, anxiety, rage and/or frustration during the processes of capture and transport for purposes, such as relocation, re-homing or permanent penning [e.g., (2, 15, 34, 92)]. Other unpleasant experiences, such as pain or exhaustion may arise due to physical injury or capture myopathy [e.g., (177, 178)].

Importantly, scientific studies now provide evidence of ongoing negative welfare impacts in animals relocated rather than humanely killed [e.g., (15, 17, 24, 139, 140)]. Other studies compare potential impacts associated with all components of lethal vs. non-lethal methods to allow holistic decision-making (18, 24). Impacts occurring after the period of capture, temporary holding and release may take the form of extreme hunger due to unfamiliarity with foraging opportunities (34), or fear and pain due to the animal's reduced ability to escape predators in the new location or because of aggression from resident conspecifics (2, 15, 140). For animals captured from the wild and brought into captivity, for example, for permanent penning or taming, there is undoubtedly a period of severe fear and anxiety as they habituate to confinement and human proximity (78, 179); some individuals never successfully acclimate, meaning such experiences likely persist to some degree [e.g., (33, 180–183)]. Disruption of social groups and restricted movement may lead to other, less well-understood unpleasant experiences, such as loneliness, frustration, boredom, depression or grief [e.g., (62, 150, 151)].

Similarly, decisions about whether to rehabilitate or promptly euthanize “rescued” wildlife should not be evaluated only in terms of the conservation status of the species and the genetic merit of the individual, but also by considering the potential for significant and/or chronic unpleasant experiences, such as pain, sickness and fear, both during and after the rehabilitation process [e.g., (30, 95, 97, 184–186)]. In both cases, the potential for longer-term negative welfare impacts is often not formally evaluated in conservation decisions, and, in any case, the significance of such impacts for the animal itself may be overwhelmed by public sentiment about the value of sustaining life at any cost over a humane death [e.g., (96, 187)].

## Whether and/or how to consider information in development of policy and legislation

As well as influencing current conservation decision-making, research and practice, the conceptual basis of welfare may also influence development of policies, guidelines and laws that, in turn, guide future practice. In particular, emphasizing that some animals experience unpleasant (and pleasant) states which affect their welfare highlights the significance of legislative discrepancies and the limitations of using survival or biological function to infer welfare in conservation and other policies and guidelines.

In NZ's Animal Welfare Act 1999 (170) and Codes of Welfare enacted under that Act, persons in charge of wild animals held for the purposes of exhibition, containment or rehabilitation are obligated to meet the animals' physical, health and behavioral needs and to act to avoid or alleviate any unnecessary or unreasonable pain or distress [e.g., (188)]. Other wild animals are variously recognized and treated under the law (see below). Although there is general reference in the law to one specific unpleasant experience, i.e., pain, and an amalgamation of others under the appellation “distress” (54), the importance of unpleasant experiences for animal welfare is not explicitly articulated, which may encourage emphasis on physical state, the limits of which have been discussed above. The importance of interpreting observable or measurable indicators as reflective of animals' mental experiences in the legal context has recently been exemplified in a number of successful legal prosecutions for animal welfare offenses in Canada and the UK (168, 189, 190).

For free-living wild animals or animals living in a wild state (i.e., feral domestic animals), there exist incongruities among NZ laws or even among sections of the same Act that appear to facilitate de-prioritization of animals' mental experiences in certain contexts (41). These “exemptions” to general requirements to safeguard animal welfare become more difficult to defend for economic, conservation or practical reasons if the experiences of the animals themselves are central to our collective conception of welfare. To illustrate, under Section 30A of the NZ Animal Welfare Act, “a person commits an offense who wilfully ill-treats a wild animal.” Ill-treatment is defined as “causing the animal to suffer pain or distress that, in its kind or degree, is unreasonable or unnecessary.” However, it is legally acceptable to purposefully use control methods scientifically demonstrated to be relatively less humane than existing alternatives for some sentient wild animals, either because of their classification as “pests” or because it is “generally accepted” to treat them in that particular way (170). Some of these exemptions relate to fulfillment of the purposes of other acts, such as the Conservation Act 1987 (191) and the Biosecurity Act 1993 (192) (Animal Welfare Amendment Act (No.2) 2015 (193) subsection 30A4) or the Animal Welfare Act, Section 181, relating to the Agricultural Compounds and Veterinary Medicines Act 1997 (194), when the activity involves the use of any substance for direct management or eradication of vertebrate pests. Nonetheless, the question arises: “is the suffering caused to these wild animals ‘necessary'?” (41, 52, 195).

There are also examples of animals of the same species being treated differently under the law when they are classified differently for human purposes. For example, feral cats (*Felis domesticus*) are designated as pests and are thus exempt from certain welfare protections under various NZ laws, as described above. In contrast, owned cats of the same species (*Felis domesticus*) and cats used for the purposes of research, which presumably have the same biological capacity for unpleasant experiences that compromise their welfare, are much more strongly protected under the NZ Animal Welfare Act. These categorizations and legal exemptions serve to reinforce existing species and contextual biases (41, 74) and are likely to stymie progressive development of more humane methods for managing wild populations, both of which are detrimental to wild animal welfare overall.

## Examples of “conservation welfare” in the zoo community

As noted above, collaborative research and practice among conservation and animal welfare scientists occur only sporadically. Explicit and deliberate evaluations of welfare occur in some specific areas of biological conservation, particularly in context of research involving wild animals, when approval from a regulatory body is required, and for animals kept in zoos.

Zoos arguably play roles in *ex situ* conservation by providing genetic repositories for threatened and endemic species and by educating the public about animals and conservation [e.g., (196–198)] [but cf e.g., (199)]. In these roles, the zoo community is demonstrating a commitment to “Conservation Welfare” in various ways, most notably by adopting a contemporary characterization of animal welfare and scientific principles and methods of assessment to guide zoo design and practices [e.g., (115, 200–204)]. Two key examples are the World Association of Zoos and Aquariums Animal Welfare Strategy (46) and the Zoo and Aquarium Association (Australasia) members' accreditation programme [(205, 206); n.d.]. Both documents are based on a characterization of animal welfare and assessment framework reflecting the centrality of animals' mental experiences. To become accredited ZAA members, Australasian zoos and aquaria must demonstrate the ways in which they provide care and husbandry practices and habitats designed to minimize unpleasant experiences and maximize opportunities for animals to have positive experiences [(205, 206); n.d.].

For various reasons, this approach may be easier and also more pressing for the zoo community to action than for biologists working in other areas of conservation practice. Maintaining public support is of primary importance for the continued existence of zoos, and zoo practices, including those reflecting a commitment to animal welfare, are under increasing public scrutiny (204). Zoo scientists are able to evaluate welfare at the level of the individual animal over time and are able to collect much more detailed data than field biologists usually can (10, 75). Increasingly, this kind of information and a focus on animals' mental experiences is guiding habitat design [e.g., (133, 207)] and the evolution of zoo policies and guidelines (116), ZAA's Animal Welfare Position Statement (205) and is being given greater weight in conservation decision-making in the zoo community [e.g., Periera (208) “Tiger returned to SF zoo after transfer to Sacramento made her homesick”; Anon (209) “Zoo pays tribute to much-loved lions”; Johnston (210) “Auckland zoo puts down ‘unhappy and agitated' gibbon”]. Individual zoo organizations, and increasingly the zoo community as a whole, are showing leadership in this regard, and there is great potential for zoo biologists and welfare scientists to collaborate more closely with their field research colleagues to optimize policies and practices to better achieve both welfare and conservation goals more broadly [e.g., (211, 212)].

## Concluding remarks: a future of conservation welfare

To address some of the challenges identified above, the establishment of a new discipline of “Conservation Welfare” is recommended. Its major role would be to reveal key synergies between the sciences of conservation and animal welfare with the aim of providing an integrated foundation upon which the two could interact constructively to further the objectives of both. Finding common ground has apparently been hindered thus far by notions that these are competing disciplines or schools of thought, or even ideologies. In part, this has been due to different ways members of the two disciplines have understood animal welfare, with conservation scientists generally emphasizing “fitness” and welfare scientists “feelings,” as illustrated here. This dichotomy has led to apparently incompatible views on the nature and significance of animal welfare impacts and the related implications for wildlife policy and management. Some of these difficulties have been considered here, and these observations raise the question of how this impasse can be resolved.

It is concluded that to make progress scientists in both disciplines will need to arrive at compatible understandings of animal welfare; in other words, it will behoove both groups to use a common language when considering welfare matters in the conservation context. Thus, instead of reinforcing the existing “fitness” *or* “feelings” dichotomy, cross-disciplinary progress may be achieved by recognizing the scientifically current and widely accepted animal welfare conceptual framework that integrates these two elements as dynamically interacting components within animals, i.e., that animals embody a “fitness” *and* “feelings” unity. Understanding this unity underpins the conceptual foundations of animal welfare and rigorous and robust science-based methods used to assess animal welfare impacts in circumstances that compromise and/or enhance welfare.

It is still necessary to consider various matters in more detail than was possible here. They include: what the precise implications will be for informed decision-making in the conservation arena; what will constitute humane conservation practices and/or management; how public perceptions and values will evolve to interact with welfare and conservation decision-making and practice; and how high standards of individual and/or group animal welfare can be monitored and achieved practically in conservation biology whilst most effectively meeting both conservation and animal welfare objectives.

## Author contributions

NB and DM designed and facilitated the workshop during which the ideas expressed in this paper were collectively generated by all authors. NB wrote the first draft of the paper and all other authors provided critical review of the drafts.

### Conflict of interest statement

The authors declare that the research was conducted in the absence of any commercial or financial relationships that could be construed as a potential conflict of interest.
